# (2*E*)-3-(6-Meth­oxy­naphthalen-2-yl)-1-[4-(methyl­sulfan­yl)phen­yl]prop-2-en-1-one

**DOI:** 10.1107/S1600536812028930

**Published:** 2012-06-30

**Authors:** Hoong-Kun Fun, Tze Shyang Chia, Mahesh Padaki, Arun M. Isloor, A. F. Ismail

**Affiliations:** a X-ray Crystallography Unit, School of Physics, Universiti Sains Malaysia, 11800 USM, Penang, Malaysia; b Membrane Technology Laboratory, Department of Chemistry, National Institute of Technology-Karnataka, Surathkal, Mangalore 575 025, India; c Advanced Membrane Science and Technology Centre (AMTEC), Universiti Teknologi Malaysia (UTM), Skudai, Johor Bahru, Malaysia

## Abstract

The asymmetric unit of the title compound, C_21_H_18_O_2_S, consists of two crystallographically independent mol­ecules (*A* and *B*). The mol­ecules exist in a *trans* conformation with respect to the central C=C bond. The naphthalene ring system makes dihedral angles of 51.62 (12) (mol­ecule *A*) and 52.69 (12)° (mol­ecule *B*) with the benzene ring. In mol­ecule *A*, the prop-2-en-1-one group forms dihedral angles of 22.84 (15) and 29.02 (12)° with the adjacent naphthalene ring system and benzene ring, respectively, whereas the corresponding angles are 30.04 (12) and 23.33 (12)° in mol­ecule *B*. In the crystal, mol­ecules are linked by inter­molecular C—H⋯O hydrogen bonds into head-to-tail chains along the *a* axis. The crystal packing also features C—H⋯π inter­actions. The crystal studied was a pseudo-merohedral twin with twin law (100 0-10 00-1) and a refined component ratio of 0.6103 (16):0.3897 (16).

## Related literature

For the preparation and applications of chalcones, see: Mori *et al.* (2003 ▶); Kumar *et al.* (2006 ▶); Amir *et al.* (2008 ▶); Atwal *et al.* (1990 ▶). For a related structure, see: Kobkeatthawin *et al.* (2011 ▶). For reference bond lengths, see: Allen *et al.* (1987 ▶). For stability of the temperature controller used in the data collection, see: Cosier & Glazer (1986 ▶).
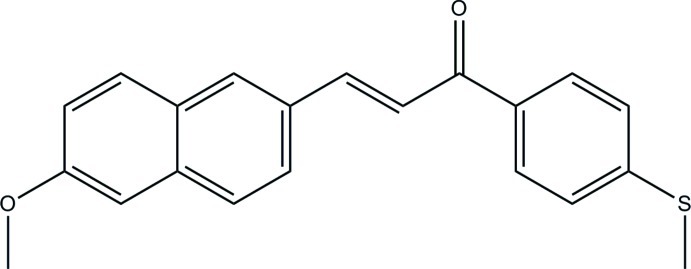


## Experimental

### 

#### Crystal data

C_21_H_18_O_2_S
*M* *_r_* = 334.41
Monoclinic, 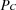

*a* = 18.6118 (14) Å
*b* = 15.0510 (12) Å
*c* = 5.9227 (5) Å
β = 90.0005 (15)°
*V* = 1659.1 (2) Å^3^
*Z* = 4
Mo *K*α radiation
μ = 0.21 mm^−1^
*T* = 100 K
0.45 × 0.10 × 0.09 mm

#### Data collection

Bruker APEX DUO CCD area-detector diffractometer
Absorption correction: multi-scan (*SADABS*; Bruker, 2009 ▶) *T*_min_ = 0.913, *T*_max_ = 0.982
18668 measured reflections
8757 independent reflections
8185 reflections with *I* > 2σ(*I*)
*R*_int_ = 0.041

#### Refinement

*R*[*F* ^2^ > 2σ(*F* ^2^)] = 0.055
*wR*(*F* ^2^) = 0.136
*S* = 1.05
8757 reflections
438 parameters
2 restraints
H-atom parameters constrained
Δρ_max_ = 0.65 e Å^−3^
Δρ_min_ = −0.37 e Å^−3^
Absolute structure: Flack (1983 ▶), 3893 Friedel pairs
Flack parameter: 0.24 (8)

### 

Data collection: *APEX2* (Bruker, 2009 ▶); cell refinement: *SAINT* (Bruker, 2009 ▶); data reduction: *SAINT*; program(s) used to solve structure: *SHELXTL* (Sheldrick, 2008 ▶); program(s) used to refine structure: *SHELXTL*; molecular graphics: *SHELXTL*; software used to prepare material for publication: *SHELXTL* and *PLATON* (Spek, 2009 ▶).

## Supplementary Material

Crystal structure: contains datablock(s) global, I. DOI: 10.1107/S1600536812028930/rz2777sup1.cif

Structure factors: contains datablock(s) I. DOI: 10.1107/S1600536812028930/rz2777Isup2.hkl

Supplementary material file. DOI: 10.1107/S1600536812028930/rz2777Isup3.cml

Additional supplementary materials:  crystallographic information; 3D view; checkCIF report

## Figures and Tables

**Table 1:** Hydrogen-bond geometry (Å, °) *Cg*1, *Cg*2 and *Cg*3 are the centroids of the C1*A*/C2*A*/C7*A*–C10*A*, C14*A*–C19*A* and C1*B*/C2*B*/C7*B*–C10*B* rings, respectively

*D*—H⋯*A*	*D*—H	H⋯*A*	*D*⋯*A*	*D*—H⋯*A*
C20*A*—H20*A*⋯O1*A* ^i^	0.96	2.44	3.381 (5)	165
C20*B*—H20*D*⋯O1*B* ^i^	0.96	2.39	3.252 (5)	149
C8*A*—H8*AA*⋯*Cg*1^ii^	0.93	2.84	3.565 (3)	136
C3*B*—H3*BA*⋯*Cg*2^iii^	0.93	2.74	3.479 (3)	137
C8*B*—H8*BA*⋯*Cg*3^iv^	0.93	2.78	3.494 (3)	134
C20*A*—H20*B*⋯*Cg*3^v^	0.96	2.63	3.481 (5)	148

Symmetry codes: (i) 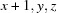; (ii) 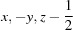; (iii) 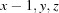; (iv) 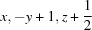; (v) 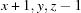.

**Fig. 1.**
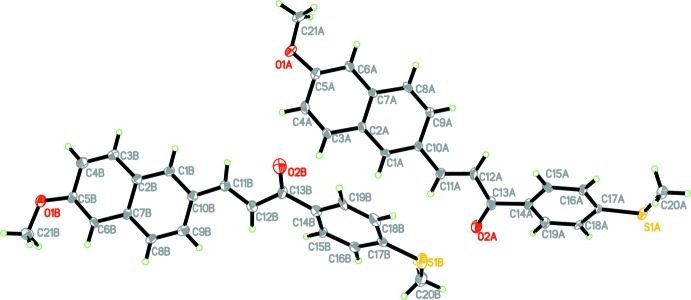
The molecular structure of the title compound with atom labels and 50% probability displacement ellipsoids.

**Fig. 2.**
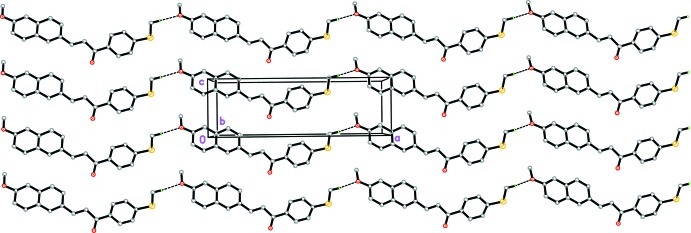
The crystal packing of the title compound. Hydrogen atoms not involved in hydrogen bonding (dashed lines) have been omitted for clarity.

## supplementary crystallographic information

### Comment

Chalcones are unsaturated ketones containing the reactive ketoethylenic group —CO—CH═CH—. These compounds are coloured due to the presence of the chromophore —CO—CH═CH—, and depends on the presence of other auxochromes. Several methods are available for the preparation of chalcones (Mori *et al.*, 2003; Kumar *et al.*, 2006). The most convenient method is the Claisen-Schimdt condensation of equimolar quantities of arylmethylketone with aryl aldehyde in the presence of alcoholic alkali (Amir *et al.*, 2008). Chalcones are used to synthesize several derivatives like cyanopyridines, pyrazolines, isoxazoles and pyrimidines with different heterocyclic ring systems (Atwal *et al.*, 1990). In view of the importance of chalcones, the title compound was synthesized and its crystal structure is reported herein.

The asymmetric unit of the title compound (Fig. 1) consists of two crystallographically independent molecules (*A* and *B*). The molecules exist in *trans* configuration with respect to the central C11═C12 bond. The naphthalene ring system [C1–C10; maximum deviations = 0.037 (3) and 0.041 (3) Å at atom C4 for both molecules] makes dihedral angles of 51.62 (12)° [molecule *A*] and 52.69 (12)° [molecule *B*] with the C14–C19 benzene ring. The prop-2-en-1-one group [maximum deviations = 0.0426 (23) Å at atom C13A and 0.0846 (21) Å at atom C13B] forms dihedral angles of 22.84 (15) and 29.02 (12)° with the adjacent naphthalene ring system and benzene ring, respectively in molecules *A*, whereas the corresponding angles are 30.04 (12) and 23.33 (12)° in molecule *B*. Bond lengths (Allen *et al.*, 1987) and angles are within normal ranges and are comparable to those found in a related structure (Kobkeatthawin *et al.*, 2011).

In the crystal (Fig. 2), molecules are linked by intermolecular C20A—H20A···O1A and C20B—H20D···O1B hydrogen bonds into chains in head-to-tail fashion, propagating along the *a* axis. The crystal packing is further stabilized by the C—H···π interactions (Table 1), involving *Cg*1, *Cg*2 and *Cg*3 which are the centroids of C1A/C2A/C7A–C10A, C14A–C19A and C1B/C2B/C7B–C10B rings, respectively.

### Experimental

To a thoroughly stirred solution of 6-methoxy-2-naphthaldehyde (0.5 g, 10 mmol) and 1-[4-(methylsulfanyl)phenyl]ethanone (1.66 g, 10 mmol) in 5 ml methanol, 0.5 ml of 40% NaOH solution was added. The reaction mixture was stirred overnight and the solid separated was collected by filtration. The product obtained was recrystallized from methanol. Yield: 2.65 g, 79.3%. M.p. 459–461 K.

### Refinement

All H atoms were positioned geometrically [C—H = 0.93 and 0.96 Å] and refined using a riding model with *U*_iso_(H) = 1.2 or 1.5*U*_eq_(C). A rotating group model was applied to the methyl groups. The crystal was a pseudo-merohedral twin with twin law (100 010 001) and BASF of 0.3897 (16).

### Crystal data

**Table:**

C_21_H_18_O_2_S	*F*(000) = 704
*M**_r_* = 334.41	*D*_x_ = 1.339 Mg m^−^^3^
Monoclinic, *P**c*	Mo *K*α radiation, λ = 0.71073 Å
Hall symbol: P -2yc	Cell parameters from 6635 reflections
*a* = 18.6118 (14) Å	θ = 2.6–29.9°
*b* = 15.0510 (12) Å	µ = 0.21 mm^−^^1^
*c* = 5.9227 (5) Å	*T* = 100 K
β = 90.0005 (15)°	Needle, yellow
*V* = 1659.1 (2) Å^3^	0.45 × 0.10 × 0.09 mm
*Z* = 4	

### Data collection

**Table:**

Bruker APEX DUO CCD area-detector diffractometer	8757 independent reflections
Radiation source: fine-focus sealed tube	8185 reflections with *I* > 2σ(*I*)
Graphite monochromator	*R*_int_ = 0.041
φ and ω scans	θ_max_ = 30.1°, θ_min_ = 1.7°
Absorption correction: multi-scan (*SADABS*; Bruker, 2009)	*h* = −26→26
*T*_min_ = 0.913, *T*_max_ = 0.982	*k* = −21→20
18668 measured reflections	*l* = −8→8

### Refinement

**Table:**

Refinement on *F*^2^	Secondary atom site location: difference Fourier map
Least-squares matrix: full	Hydrogen site location: inferred from neighbouring sites
*R*[*F*^2^ > 2σ(*F*^2^)] = 0.055	H-atom parameters constrained
*wR*(*F*^2^) = 0.136	*w* = 1/[σ^2^(*F*_o_^2^) + (0.0704*P*)^2^] where *P* = (*F*_o_^2^ + 2*F*_c_^2^)/3
*S* = 1.05	(Δ/σ)_max_ = 0.001
8757 reflections	Δρ_max_ = 0.65 e Å^−^^3^
438 parameters	Δρ_min_ = −0.37 e Å^−^^3^
2 restraints	Absolute structure: Flack (1983), 3893 Friedel pairs
Primary atom site location: structure-invariant direct methods	Flack parameter: 0.24 (8)

### Special details

**Table:**

Experimental. The crystal was placed in the cold stream of an Oxford Cryosystems Cobra open-flow nitrogen cryostat (Cosier & Glazer, 1986) operating at 100.0 (1) K.
Geometry. All e.s.d.'s (except the e.s.d. in the dihedral angle between two l.s. planes) are estimated using the full covariance matrix. The cell e.s.d.'s are taken into account individually in the estimation of e.s.d.'s in distances, angles and torsion angles; correlations between e.s.d.'s in cell parameters are only used when they are defined by crystal symmetry. An approximate (isotropic) treatment of cell e.s.d.'s is used for estimating e.s.d.'s involving l.s. planes.
Refinement. Refinement of *F*^2^ against ALL reflections. The weighted *R*-factor *wR* and goodness of fit *S* are based on *F*^2^, conventional *R*-factors *R* are based on *F*, with *F* set to zero for negative *F*^2^. The threshold expression of *F*^2^ > σ(*F*^2^) is used only for calculating *R*-factors(gt) *etc*. and is not relevant to the choice of reflections for refinement. *R*-factors based on *F*^2^ are statistically about twice as large as those based on *F*, and *R*- factors based on ALL data will be even larger.

### Fractional atomic coordinates and isotropic or equivalent isotropic displacement parameters (Å^2^)

**Table:**

	*x*	*y*	*z*	*U*_iso_*/*U*_eq_	
S1A	1.08822 (5)	0.10393 (6)	0.36728 (17)	0.02342 (18)	
O1A	0.26820 (13)	0.12565 (17)	−0.1262 (6)	0.0267 (5)	
O2A	0.75451 (14)	0.1316 (2)	0.7822 (5)	0.0350 (6)	
C1A	0.52042 (16)	0.16767 (19)	0.3703 (6)	0.0150 (5)	
H1AA	0.5183	0.1960	0.5096	0.018*	
C2A	0.45726 (17)	0.15987 (19)	0.2382 (6)	0.0155 (6)	
C3A	0.38979 (15)	0.19520 (18)	0.3088 (6)	0.0173 (6)	
H3AA	0.3865	0.2254	0.4455	0.021*	
C4A	0.33029 (17)	0.1853 (2)	0.1788 (7)	0.0219 (7)	
H4AA	0.2873	0.2113	0.2245	0.026*	
C5A	0.33241 (18)	0.1362 (2)	−0.0252 (7)	0.0191 (7)	
C6A	0.39676 (18)	0.1015 (2)	−0.1018 (7)	0.0180 (6)	
H6AA	0.3985	0.0702	−0.2370	0.022*	
C7A	0.45972 (17)	0.11400 (19)	0.0271 (6)	0.0136 (6)	
C8A	0.52828 (17)	0.0818 (2)	−0.0451 (6)	0.0177 (6)	
H8AA	0.5316	0.0531	−0.1837	0.021*	
C9A	0.58886 (19)	0.09160 (19)	0.0813 (6)	0.0172 (6)	
H9AA	0.6327	0.0707	0.0280	0.021*	
C10A	0.58450 (17)	0.13411 (19)	0.2960 (6)	0.0146 (5)	
C11A	0.64673 (18)	0.1402 (2)	0.4461 (6)	0.0194 (6)	
H11A	0.6372	0.1588	0.5928	0.023*	
C12A	0.71575 (16)	0.1225 (2)	0.3995 (7)	0.0193 (7)	
H12A	0.7293	0.1082	0.2528	0.023*	
C13A	0.77081 (19)	0.1256 (2)	0.5813 (7)	0.0226 (7)	
C14A	0.84832 (17)	0.1204 (2)	0.5130 (7)	0.0167 (6)	
C15A	0.87291 (18)	0.1513 (2)	0.3048 (6)	0.0177 (6)	
H15A	0.8403	0.1730	0.1994	0.021*	
C16A	0.94659 (16)	0.14992 (19)	0.2532 (6)	0.0157 (6)	
H16A	0.9633	0.1726	0.1169	0.019*	
C17A	0.99451 (15)	0.11367 (19)	0.4108 (6)	0.0137 (6)	
C18A	0.96989 (16)	0.08086 (19)	0.6188 (6)	0.0163 (6)	
H18A	1.0019	0.0559	0.7215	0.020*	
C19A	0.89735 (18)	0.0861 (2)	0.6689 (7)	0.0182 (6)	
H19A	0.8810	0.0664	0.8086	0.022*	
C20A	1.1033 (2)	0.1573 (3)	0.0992 (7)	0.0312 (9)	
H20A	1.1534	0.1538	0.0613	0.047*	
H20B	1.0891	0.2185	0.1087	0.047*	
H20C	1.0755	0.1281	−0.0153	0.047*	
C21A	0.2639 (2)	0.0665 (3)	−0.3144 (7)	0.0290 (8)	
H21A	0.2150	0.0621	−0.3638	0.044*	
H21B	0.2809	0.0088	−0.2703	0.044*	
H21C	0.2931	0.0888	−0.4354	0.044*	
S1B	0.65875 (5)	0.38034 (6)	0.7749 (2)	0.0290 (2)	
O1B	−0.17124 (13)	0.37693 (16)	1.1700 (5)	0.0232 (5)	
O2B	0.32634 (15)	0.40237 (17)	0.3454 (6)	0.0281 (6)	
C1B	0.08998 (19)	0.33729 (19)	0.7171 (6)	0.0173 (6)	
H1BA	0.0896	0.3105	0.5756	0.021*	
C2B	0.02541 (17)	0.34493 (18)	0.8358 (6)	0.0171 (6)	
C3B	−0.04127 (17)	0.31198 (19)	0.7549 (6)	0.0191 (6)	
H3BA	−0.0420	0.2826	0.6168	0.023*	
C4B	−0.10425 (17)	0.3214 (2)	0.8700 (7)	0.0207 (6)	
H4BA	−0.1465	0.2968	0.8142	0.025*	
C5B	−0.10433 (18)	0.3700 (2)	1.0789 (6)	0.0183 (6)	
C6B	−0.04156 (17)	0.4025 (2)	1.1700 (6)	0.0163 (6)	
H6BA	−0.0422	0.4323	1.3076	0.020*	
C7B	0.02500 (17)	0.3900 (2)	1.0507 (6)	0.0155 (6)	
C8B	0.09134 (18)	0.41933 (19)	1.1407 (6)	0.0175 (6)	
H8BA	0.0924	0.4465	1.2817	0.021*	
C9B	0.1544 (2)	0.4080 (2)	1.0217 (7)	0.0207 (7)	
H9BA	0.1975	0.4267	1.0855	0.025*	
C10B	0.15495 (18)	0.36844 (18)	0.8037 (7)	0.0174 (7)	
C11B	0.21800 (18)	0.3661 (2)	0.6598 (6)	0.0174 (6)	
H11B	0.2111	0.3475	0.5117	0.021*	
C12B	0.28628 (19)	0.3885 (2)	0.7199 (7)	0.0224 (7)	
H12B	0.2974	0.4005	0.8699	0.027*	
C13B	0.34251 (18)	0.3934 (2)	0.5438 (7)	0.0185 (7)	
C14B	0.41983 (19)	0.38928 (19)	0.6142 (7)	0.0174 (6)	
C15B	0.44145 (15)	0.35310 (19)	0.8180 (6)	0.0142 (6)	
H15B	0.4073	0.3326	0.9201	0.017*	
C16B	0.51400 (18)	0.3474 (2)	0.8703 (7)	0.0202 (6)	
H16B	0.5279	0.3219	1.0066	0.024*	
C17B	0.56853 (17)	0.38018 (18)	0.7174 (6)	0.0152 (6)	
C18B	0.5429 (2)	0.4159 (2)	0.5152 (7)	0.0220 (7)	
H18B	0.5763	0.4383	0.4131	0.026*	
C19B	0.47186 (17)	0.4199 (2)	0.4584 (6)	0.0169 (6)	
H19B	0.4579	0.4426	0.3190	0.020*	
C20B	0.6682 (2)	0.3138 (3)	1.0244 (8)	0.0311 (8)	
H20D	0.7182	0.3083	1.0618	0.047*	
H20E	0.6484	0.2559	0.9977	0.047*	
H20F	0.6432	0.3416	1.1473	0.047*	
C21B	−0.17812 (19)	0.4323 (2)	1.3649 (7)	0.0261 (7)	
H21D	−0.2281	0.4428	1.3952	0.039*	
H21E	−0.1544	0.4879	1.3381	0.039*	
H21F	−0.1565	0.4033	1.4924	0.039*	

### Atomic displacement parameters (Å^2^)

**Table:**

	*U*^11^	*U*^22^	*U*^33^	*U*^12^	*U*^13^	*U*^23^
S1A	0.0118 (3)	0.0298 (4)	0.0287 (5)	−0.0011 (3)	−0.0032 (4)	−0.0013 (4)
O1A	0.0175 (11)	0.0359 (12)	0.0267 (14)	0.0022 (9)	−0.0083 (13)	−0.0057 (13)
O2A	0.0178 (12)	0.0736 (19)	0.0135 (12)	0.0016 (12)	−0.0024 (12)	0.0005 (15)
C1A	0.0163 (12)	0.0190 (12)	0.0097 (13)	0.0026 (10)	−0.0015 (13)	−0.0023 (12)
C2A	0.0128 (12)	0.0167 (12)	0.0169 (15)	0.0035 (10)	0.0019 (13)	−0.0011 (12)
C3A	0.0143 (12)	0.0174 (11)	0.0204 (15)	0.0013 (9)	0.0016 (12)	−0.0048 (12)
C4A	0.0143 (13)	0.0220 (14)	0.0293 (18)	0.0047 (11)	0.0030 (14)	−0.0020 (14)
C5A	0.0151 (15)	0.0188 (14)	0.0235 (17)	−0.0001 (11)	−0.0025 (14)	0.0009 (13)
C6A	0.0159 (14)	0.0218 (14)	0.0163 (16)	0.0031 (11)	0.0036 (14)	0.0000 (12)
C7A	0.0138 (14)	0.0167 (14)	0.0104 (13)	0.0009 (10)	0.0015 (13)	0.0008 (11)
C8A	0.0197 (15)	0.0229 (14)	0.0104 (13)	0.0042 (11)	0.0055 (13)	0.0012 (11)
C9A	0.0130 (12)	0.0195 (13)	0.0191 (15)	0.0017 (11)	0.0011 (14)	−0.0014 (12)
C10A	0.0117 (12)	0.0193 (12)	0.0127 (13)	0.0048 (10)	−0.0006 (14)	0.0006 (12)
C11A	0.0147 (14)	0.0265 (15)	0.0169 (16)	0.0008 (12)	−0.0010 (14)	0.0022 (13)
C12A	0.0091 (12)	0.0290 (15)	0.0198 (18)	0.0014 (10)	0.0016 (13)	0.0046 (14)
C13A	0.0147 (15)	0.0344 (17)	0.0188 (17)	0.0007 (13)	0.0032 (14)	0.0084 (14)
C14A	0.0126 (14)	0.0202 (14)	0.0173 (16)	0.0007 (11)	−0.0016 (13)	0.0030 (12)
C15A	0.0199 (14)	0.0228 (14)	0.0105 (14)	0.0060 (11)	0.0015 (13)	0.0012 (12)
C16A	0.0112 (12)	0.0165 (12)	0.0192 (16)	0.0007 (9)	0.0001 (13)	−0.0004 (12)
C17A	0.0070 (11)	0.0168 (12)	0.0173 (17)	−0.0017 (9)	0.0014 (12)	−0.0055 (11)
C18A	0.0131 (13)	0.0146 (12)	0.0213 (17)	−0.0036 (10)	−0.0057 (13)	−0.0004 (11)
C19A	0.0167 (14)	0.0179 (13)	0.0201 (15)	−0.0007 (10)	0.0010 (14)	0.0034 (12)
C20A	0.0181 (16)	0.048 (2)	0.028 (2)	−0.0047 (14)	0.0022 (16)	−0.0040 (18)
C21A	0.0246 (17)	0.0407 (19)	0.0218 (17)	−0.0028 (15)	−0.0100 (16)	−0.0025 (16)
S1B	0.0183 (4)	0.0299 (4)	0.0388 (6)	−0.0019 (3)	0.0068 (4)	0.0041 (4)
O1B	0.0141 (11)	0.0307 (12)	0.0247 (13)	−0.0029 (9)	0.0019 (11)	−0.0019 (11)
O2B	0.0273 (13)	0.0315 (12)	0.0254 (15)	0.0045 (10)	0.0019 (13)	0.0080 (12)
C1B	0.0177 (13)	0.0218 (13)	0.0125 (14)	−0.0009 (12)	−0.0021 (13)	0.0003 (11)
C2B	0.0186 (14)	0.0122 (11)	0.0204 (17)	0.0012 (9)	−0.0028 (14)	−0.0003 (11)
C3B	0.0189 (13)	0.0196 (12)	0.0187 (15)	−0.0035 (10)	−0.0036 (14)	−0.0001 (13)
C4B	0.0214 (14)	0.0189 (13)	0.0219 (15)	−0.0035 (10)	−0.0008 (16)	0.0003 (13)
C5B	0.0110 (13)	0.0225 (14)	0.0214 (17)	−0.0030 (11)	−0.0009 (14)	0.0021 (13)
C6B	0.0151 (13)	0.0182 (13)	0.0156 (14)	0.0030 (11)	0.0040 (13)	−0.0014 (12)
C7B	0.0117 (14)	0.0204 (14)	0.0145 (15)	0.0002 (10)	−0.0008 (13)	0.0050 (12)
C8B	0.0177 (13)	0.0172 (13)	0.0175 (14)	−0.0005 (11)	−0.0052 (14)	0.0012 (11)
C9B	0.0228 (17)	0.0183 (13)	0.0211 (17)	−0.0029 (12)	−0.0049 (16)	0.0002 (13)
C10B	0.0170 (13)	0.0121 (11)	0.0231 (19)	−0.0016 (10)	−0.0014 (15)	−0.0009 (12)
C11B	0.0176 (14)	0.0204 (14)	0.0141 (14)	0.0015 (11)	−0.0002 (13)	0.0023 (12)
C12B	0.0199 (16)	0.0271 (16)	0.0203 (18)	0.0008 (12)	0.0022 (15)	−0.0018 (13)
C13B	0.0133 (15)	0.0231 (15)	0.0192 (17)	0.0001 (11)	−0.0005 (13)	0.0052 (13)
C14B	0.0193 (14)	0.0121 (12)	0.0207 (17)	−0.0030 (10)	0.0039 (13)	−0.0002 (11)
C15B	0.0106 (12)	0.0181 (12)	0.0140 (15)	−0.0035 (9)	0.0017 (11)	0.0020 (11)
C16B	0.0203 (14)	0.0214 (13)	0.0189 (16)	−0.0018 (11)	0.0029 (15)	−0.0034 (14)
C17B	0.0187 (14)	0.0095 (11)	0.0176 (15)	0.0055 (9)	−0.0008 (12)	−0.0038 (10)
C18B	0.0258 (16)	0.0164 (13)	0.0237 (18)	0.0012 (12)	0.0100 (16)	−0.0005 (13)
C19B	0.0188 (14)	0.0159 (13)	0.0161 (14)	0.0024 (10)	0.0094 (13)	0.0018 (11)
C20B	0.0211 (16)	0.0379 (19)	0.034 (2)	0.0000 (14)	−0.0004 (17)	0.0001 (17)
C21B	0.0251 (15)	0.0360 (17)	0.0171 (15)	−0.0016 (13)	0.0050 (16)	−0.0020 (16)

### Geometric parameters (Å, º)

**Table:**

S1A—C17A	1.769 (3)	S1B—C17B	1.713 (3)
S1A—C20A	1.802 (5)	S1B—C20B	1.794 (5)
O1A—C5A	1.346 (4)	O1B—C5B	1.361 (4)
O1A—C21A	1.429 (5)	O1B—C21B	1.429 (5)
O2A—C13A	1.231 (5)	O2B—C13B	1.220 (5)
C1A—C10A	1.368 (4)	C1B—C10B	1.395 (5)
C1A—C2A	1.417 (4)	C1B—C2B	1.397 (5)
C1A—H1AA	0.9300	C1B—H1BA	0.9300
C2A—C3A	1.426 (4)	C2B—C3B	1.420 (4)
C2A—C7A	1.429 (5)	C2B—C7B	1.443 (5)
C3A—C4A	1.357 (5)	C3B—C4B	1.364 (5)
C3A—H3AA	0.9300	C3B—H3BA	0.9300
C4A—C5A	1.418 (5)	C4B—C5B	1.437 (5)
C4A—H4AA	0.9300	C4B—H4BA	0.9300
C5A—C6A	1.383 (5)	C5B—C6B	1.377 (5)
C6A—C7A	1.411 (5)	C6B—C7B	1.439 (5)
C6A—H6AA	0.9300	C6B—H6BA	0.9300
C7A—C8A	1.430 (4)	C7B—C8B	1.415 (5)
C8A—C9A	1.361 (5)	C8B—C9B	1.379 (5)
C8A—H8AA	0.9300	C8B—H8BA	0.9300
C9A—C10A	1.426 (5)	C9B—C10B	1.422 (5)
C9A—H9AA	0.9300	C9B—H9BA	0.9300
C10A—C11A	1.463 (4)	C10B—C11B	1.451 (5)
C11A—C12A	1.341 (4)	C11B—C12B	1.362 (5)
C11A—H11A	0.9300	C11B—H11B	0.9300
C12A—C13A	1.487 (5)	C12B—C13B	1.479 (5)
C12A—H12A	0.9300	C12B—H12B	0.9300
C13A—C14A	1.500 (5)	C13B—C14B	1.500 (5)
C14A—C15A	1.395 (5)	C14B—C15B	1.384 (5)
C14A—C19A	1.397 (5)	C14B—C19B	1.415 (4)
C15A—C16A	1.405 (4)	C15B—C16B	1.388 (4)
C15A—H15A	0.9300	C15B—H15B	0.9300
C16A—C17A	1.402 (5)	C16B—C17B	1.447 (5)
C16A—H16A	0.9300	C16B—H16B	0.9300
C17A—C18A	1.404 (5)	C17B—C18B	1.396 (5)
C18A—C19A	1.385 (4)	C18B—C19B	1.366 (5)
C18A—H18A	0.9300	C18B—H18B	0.9300
C19A—H19A	0.9300	C19B—H19B	0.9300
C20A—H20A	0.9600	C20B—H20D	0.9600
C20A—H20B	0.9600	C20B—H20E	0.9600
C20A—H20C	0.9600	C20B—H20F	0.9600
C21A—H21A	0.9600	C21B—H21D	0.9600
C21A—H21B	0.9600	C21B—H21E	0.9600
C21A—H21C	0.9600	C21B—H21F	0.9600
			
C17A—S1A—C20A	104.21 (17)	C17B—S1B—C20B	105.05 (17)
C5A—O1A—C21A	118.0 (3)	C5B—O1B—C21B	116.5 (3)
C10A—C1A—C2A	121.0 (3)	C10B—C1B—C2B	122.2 (3)
C10A—C1A—H1AA	119.5	C10B—C1B—H1BA	118.9
C2A—C1A—H1AA	119.5	C2B—C1B—H1BA	118.9
C1A—C2A—C3A	122.5 (3)	C1B—C2B—C3B	123.6 (3)
C1A—C2A—C7A	119.8 (3)	C1B—C2B—C7B	119.2 (3)
C3A—C2A—C7A	117.7 (3)	C3B—C2B—C7B	117.2 (3)
C4A—C3A—C2A	120.8 (3)	C4B—C3B—C2B	123.1 (3)
C4A—C3A—H3AA	119.6	C4B—C3B—H3BA	118.4
C2A—C3A—H3AA	119.6	C2B—C3B—H3BA	118.4
C3A—C4A—C5A	121.2 (3)	C3B—C4B—C5B	119.0 (3)
C3A—C4A—H4AA	119.4	C3B—C4B—H4BA	120.5
C5A—C4A—H4AA	119.4	C5B—C4B—H4BA	120.5
O1A—C5A—C6A	125.4 (4)	O1B—C5B—C6B	126.4 (3)
O1A—C5A—C4A	114.6 (3)	O1B—C5B—C4B	112.4 (3)
C6A—C5A—C4A	120.0 (3)	C6B—C5B—C4B	121.2 (3)
C5A—C6A—C7A	119.4 (3)	C5B—C6B—C7B	119.5 (3)
C5A—C6A—H6AA	120.3	C5B—C6B—H6BA	120.3
C7A—C6A—H6AA	120.3	C7B—C6B—H6BA	120.3
C6A—C7A—C2A	120.7 (3)	C8B—C7B—C6B	121.7 (3)
C6A—C7A—C8A	122.3 (3)	C8B—C7B—C2B	118.3 (3)
C2A—C7A—C8A	117.0 (3)	C6B—C7B—C2B	120.0 (3)
C9A—C8A—C7A	122.5 (3)	C9B—C8B—C7B	120.8 (3)
C9A—C8A—H8AA	118.7	C9B—C8B—H8BA	119.6
C7A—C8A—H8AA	118.7	C7B—C8B—H8BA	119.6
C8A—C9A—C10A	119.5 (3)	C8B—C9B—C10B	121.4 (3)
C8A—C9A—H9AA	120.3	C8B—C9B—H9BA	119.3
C10A—C9A—H9AA	120.3	C10B—C9B—H9BA	119.3
C1A—C10A—C9A	120.1 (3)	C1B—C10B—C9B	117.9 (3)
C1A—C10A—C11A	118.2 (3)	C1B—C10B—C11B	118.5 (3)
C9A—C10A—C11A	121.7 (3)	C9B—C10B—C11B	123.3 (3)
C12A—C11A—C10A	128.4 (4)	C12B—C11B—C10B	126.5 (3)
C12A—C11A—H11A	115.8	C12B—C11B—H11B	116.7
C10A—C11A—H11A	115.8	C10B—C11B—H11B	116.7
C11A—C12A—C13A	120.3 (4)	C11B—C12B—C13B	119.2 (4)
C11A—C12A—H12A	119.8	C11B—C12B—H12B	120.4
C13A—C12A—H12A	119.8	C13B—C12B—H12B	120.4
O2A—C13A—C12A	122.2 (3)	O2B—C13B—C12B	120.6 (3)
O2A—C13A—C14A	120.1 (3)	O2B—C13B—C14B	120.6 (3)
C12A—C13A—C14A	117.8 (3)	C12B—C13B—C14B	118.7 (3)
C15A—C14A—C19A	119.5 (3)	C15B—C14B—C19B	119.9 (3)
C15A—C14A—C13A	122.5 (3)	C15B—C14B—C13B	122.5 (3)
C19A—C14A—C13A	118.0 (3)	C19B—C14B—C13B	117.5 (3)
C14A—C15A—C16A	120.5 (3)	C14B—C15B—C16B	120.1 (3)
C14A—C15A—H15A	119.7	C14B—C15B—H15B	119.9
C16A—C15A—H15A	119.7	C16B—C15B—H15B	119.9
C17A—C16A—C15A	118.8 (3)	C15B—C16B—C17B	121.4 (3)
C17A—C16A—H16A	120.6	C15B—C16B—H16B	119.3
C15A—C16A—H16A	120.6	C17B—C16B—H16B	119.3
C16A—C17A—C18A	120.9 (3)	C18B—C17B—C16B	115.4 (3)
C16A—C17A—S1A	124.2 (3)	C18B—C17B—S1B	120.3 (3)
C18A—C17A—S1A	114.9 (2)	C16B—C17B—S1B	124.3 (3)
C19A—C18A—C17A	119.1 (3)	C19B—C18B—C17B	124.0 (3)
C19A—C18A—H18A	120.5	C19B—C18B—H18B	118.0
C17A—C18A—H18A	120.5	C17B—C18B—H18B	118.0
C18A—C19A—C14A	121.1 (3)	C18B—C19B—C14B	119.2 (3)
C18A—C19A—H19A	119.5	C18B—C19B—H19B	120.4
C14A—C19A—H19A	119.5	C14B—C19B—H19B	120.4
S1A—C20A—H20A	109.5	S1B—C20B—H20D	109.5
S1A—C20A—H20B	109.5	S1B—C20B—H20E	109.5
H20A—C20A—H20B	109.5	H20D—C20B—H20E	109.5
S1A—C20A—H20C	109.5	S1B—C20B—H20F	109.5
H20A—C20A—H20C	109.5	H20D—C20B—H20F	109.5
H20B—C20A—H20C	109.5	H20E—C20B—H20F	109.5
O1A—C21A—H21A	109.5	O1B—C21B—H21D	109.5
O1A—C21A—H21B	109.5	O1B—C21B—H21E	109.5
H21A—C21A—H21B	109.5	H21D—C21B—H21E	109.5
O1A—C21A—H21C	109.5	O1B—C21B—H21F	109.5
H21A—C21A—H21C	109.5	H21D—C21B—H21F	109.5
H21B—C21A—H21C	109.5	H21E—C21B—H21F	109.5
			
C10A—C1A—C2A—C3A	−179.0 (3)	C10B—C1B—C2B—C3B	−178.4 (3)
C10A—C1A—C2A—C7A	1.9 (5)	C10B—C1B—C2B—C7B	2.9 (4)
C1A—C2A—C3A—C4A	−179.1 (3)	C1B—C2B—C3B—C4B	−178.6 (3)
C7A—C2A—C3A—C4A	0.0 (5)	C7B—C2B—C3B—C4B	0.2 (4)
C2A—C3A—C4A—C5A	3.0 (5)	C2B—C3B—C4B—C5B	2.6 (5)
C21A—O1A—C5A—C6A	7.0 (5)	C21B—O1B—C5B—C6B	8.0 (5)
C21A—O1A—C5A—C4A	−171.2 (3)	C21B—O1B—C5B—C4B	−173.4 (3)
C3A—C4A—C5A—O1A	174.6 (3)	C3B—C4B—C5B—O1B	177.6 (3)
C3A—C4A—C5A—C6A	−3.7 (5)	C3B—C4B—C5B—C6B	−3.7 (5)
O1A—C5A—C6A—C7A	−176.9 (3)	O1B—C5B—C6B—C7B	−179.5 (3)
C4A—C5A—C6A—C7A	1.2 (5)	C4B—C5B—C6B—C7B	1.9 (5)
C5A—C6A—C7A—C2A	1.8 (5)	C5B—C6B—C7B—C8B	−177.7 (3)
C5A—C6A—C7A—C8A	−178.4 (3)	C5B—C6B—C7B—C2B	0.9 (4)
C1A—C2A—C7A—C6A	176.7 (3)	C1B—C2B—C7B—C8B	−4.5 (4)
C3A—C2A—C7A—C6A	−2.4 (4)	C3B—C2B—C7B—C8B	176.7 (3)
C1A—C2A—C7A—C8A	−3.1 (4)	C1B—C2B—C7B—C6B	176.9 (3)
C3A—C2A—C7A—C8A	177.8 (3)	C3B—C2B—C7B—C6B	−2.0 (4)
C6A—C7A—C8A—C9A	−178.2 (3)	C6B—C7B—C8B—C9B	−178.9 (3)
C2A—C7A—C8A—C9A	1.7 (5)	C2B—C7B—C8B—C9B	2.5 (4)
C7A—C8A—C9A—C10A	1.1 (5)	C7B—C8B—C9B—C10B	1.3 (5)
C2A—C1A—C10A—C9A	0.9 (5)	C2B—C1B—C10B—C9B	0.8 (4)
C2A—C1A—C10A—C11A	−176.8 (3)	C2B—C1B—C10B—C11B	−173.7 (3)
C8A—C9A—C10A—C1A	−2.4 (5)	C8B—C9B—C10B—C1B	−3.0 (5)
C8A—C9A—C10A—C11A	175.2 (3)	C8B—C9B—C10B—C11B	171.3 (3)
C1A—C10A—C11A—C12A	−170.7 (3)	C1B—C10B—C11B—C12B	−175.7 (3)
C9A—C10A—C11A—C12A	11.6 (5)	C9B—C10B—C11B—C12B	10.1 (5)
C10A—C11A—C12A—C13A	−174.9 (3)	C10B—C11B—C12B—C13B	−172.1 (3)
C11A—C12A—C13A—O2A	10.5 (6)	C11B—C12B—C13B—O2B	20.4 (5)
C11A—C12A—C13A—C14A	−169.9 (3)	C11B—C12B—C13B—C14B	−160.9 (3)
O2A—C13A—C14A—C15A	−151.5 (4)	O2B—C13B—C14B—C15B	−159.8 (3)
C12A—C13A—C14A—C15A	28.9 (5)	C12B—C13B—C14B—C15B	21.5 (5)
O2A—C13A—C14A—C19A	26.2 (5)	O2B—C13B—C14B—C19B	17.0 (5)
C12A—C13A—C14A—C19A	−153.3 (3)	C12B—C13B—C14B—C19B	−161.7 (3)
C19A—C14A—C15A—C16A	−1.5 (5)	C19B—C14B—C15B—C16B	0.4 (4)
C13A—C14A—C15A—C16A	176.3 (3)	C13B—C14B—C15B—C16B	177.1 (3)
C14A—C15A—C16A—C17A	2.6 (5)	C14B—C15B—C16B—C17B	1.2 (5)
C15A—C16A—C17A—C18A	−1.3 (5)	C15B—C16B—C17B—C18B	−1.1 (4)
C15A—C16A—C17A—S1A	177.9 (2)	C15B—C16B—C17B—S1B	176.8 (2)
C20A—S1A—C17A—C16A	4.3 (3)	C20B—S1B—C17B—C18B	−171.2 (3)
C20A—S1A—C17A—C18A	−176.4 (2)	C20B—S1B—C17B—C16B	11.0 (3)
C16A—C17A—C18A—C19A	−1.1 (4)	C16B—C17B—C18B—C19B	−0.7 (4)
S1A—C17A—C18A—C19A	179.6 (2)	S1B—C17B—C18B—C19B	−178.7 (3)
C17A—C18A—C19A—C14A	2.3 (5)	C17B—C18B—C19B—C14B	2.2 (5)
C15A—C14A—C19A—C18A	−1.0 (5)	C15B—C14B—C19B—C18B	−2.1 (5)
C13A—C14A—C19A—C18A	−178.8 (3)	C13B—C14B—C19B—C18B	−179.0 (3)

### Hydrogen-bond geometry (Å, º)

Cg1, Cg2 and Cg3 are the centroids of the C1A/C2A/C7A–C10A, C14A–C19A and C1B/C2B/C7B–C10B rings, respectively

**Table:**

*D*—H···*A*	*D*—H	H···*A*	*D*···*A*	*D*—H···*A*
C20*A*—H20*A*···O1*A*^i^	0.96	2.44	3.381 (5)	165
C20*B*—H20*D*···O1*B*^i^	0.96	2.39	3.252 (5)	149
C8*A*—H8*AA*···*Cg*1^ii^	0.93	2.84	3.565 (3)	136
C3*B*—H3*BA*···*Cg*2^iii^	0.93	2.74	3.479 (3)	137
C8*B*—H8*BA*···*Cg*3^iv^	0.93	2.78	3.494 (3)	134
C20*A*—H20*B*···*Cg*3^v^	0.96	2.63	3.481 (5)	148

Symmetry codes: (i) *x*+1, *y*, *z*; (ii) *x*, −*y*, *z*−1/2; (iii) *x*−1, *y*, *z*; (iv) *x*, −*y*+1, *z*+1/2; (v) *x*+1, *y*, *z*−1.
